# Activated protein C upregulates ovarian cancer cell migration and promotes unclottability of the cancer cell microenvironment

**DOI:** 10.3892/or.2015.4061

**Published:** 2015-06-15

**Authors:** HAMDA ALTHAWADI, HALEMA ALFARSI, SAMAHER BESBES, SHAHSOLTAN MIRSHAHI, ELODIE DUCROS, ARASH RAFII, MARC POCARD, AMU THERWATH, JEANNETTE SORIA, MASSOUD MIRSHAHI

**Affiliations:** 1 UMR, Paris Diderot, Paris 7 University, Lariboisiere Hospital, INSERM U965, Paris, France; 2 Qatar Foundation, Weill Cornell Medical College in Qatar, Doha, Qatar

**Keywords:** ovarian cancer, protein C receptor, Rho-GTPase pathway, cancer cell migration, thrombosis, partial thromboplastin time, peritoneal fluid

## Abstract

The objective of this study was to evaluate the role of activated protein C (aPC), known to be a physiological anticoagulant, in ovarian cancer cell activation as well as in loss of clotting of cancer ascitic fluid. The effect of aPC on an ovarian cancer cell line (OVCAR-3) was tested in regards to i) cell migration and adhesion with the use of adhesion and wound healing assays as well as a droplet test; ii) protein phosphorylation, evaluated by cyto-ELISA; iii) cell cycle modification assessed by flow cytometric DNA quantification; and iv) anticoagulant activity evaluated by the prolongation of partial thromboplastin time (aPTT) of normal plasma in the presence or absence of aPC-treated ovarian cancer cells. In addition, the soluble endothelial protein C receptor (sEPCR) was quantified by ELISA in ascitic fluid of patients with ovarian cancer. Our results showed that in the OVCAR-3 aPC-induced cells i) an increase in cell migration was noted, which was inhibited when anti-endothelial protein C receptor (EPCR) was added to the culture medium and which may act via MEK-ERK and Rho-GTPase pathways; ii) an increase in threonine, and to a lesser extent tyrosine phosphorylation; iii) cell cycle activation (G1 to S/G2); and iv) a 2-3-fold prolongation of aPTT of normal plasma. In the peritoneal fluid, the sEPCR concentration was 71±23 ng/ml. In conclusion, free aPC binds to membrane EPCR in ovarian cancer cells and induces cell migration via MEK-ERK and Rho-GTPase pathways. This binding could also explain the loss of clotting of peritoneal fluids.

## Introduction

Ovarian cancer remains one of the most difficult oncologic challenges, as it is often discovered at advanced stages. It is diagnosed often with intraperitoneal dissemination of cancer cells and the presence of ascitic fluid. The cancer cells present in the intraperitoneal space often display intrinsic chemoresistance. The ascitic fluid is a complex exudative liquid known to contain various growth factors and cytokines (1). It has been shown that ascites could contribute to intraperitoneal cancer cell implantation and tumor development by promoting cell growth, invasion and drug resistance (2,3). One of the characteristics of all ascites including ovarian cancer ascites is its inability to clot. This may be due to the presence of several fibrinolytic and proteolytic enzymes such as plasminogen activators and matrix metalloproteinases that are not fully neutralized by their inhibitors (4,5). The absence of clotting of ascites in the peritoneal site favors tumor cell dissemination and their implantation on the peritoneal surface.

It was reported that endothelial protein C receptor (EPCR) binds protein C (PC) secreted by liver cells (6) and thereby inhibits the coagulation pathway by generation of activated protein C (aPC). Proteolytic cleavage of thrombin bound to thrombomodulin acts on PC to generate aPC. Endothelial protein C is an important regulator of homeostasis, in addition to its involvement in the systemic response to acute inflammation (7–11). On endothelial cells, the aPC, together with its cofactor protein S, degrades factors Va and VIIIa and thereby interferes with thrombin generation and abolishes the coagulation cascade (12). Endogenous aPC is shown to limit cancer cell extravasation through S1P-1-mediated vascular endothelium (13) and also plays a role in innate immune system (14,15).

Originally, EPCR was shown to be expressed on endothelial cells of large blood vessels but not in liver sinosuoidal and spleen endothelial cells (16). EPCR (CD201) expression is also detected in some inflammatory cells such as monocytes and neutrophils (17). Some authors claim that EPCR could be considered as a cancer stem cell marker (18). EPCR exists as membrane bound as well as free soluble endothelial protein C receptor (sEPCR) form (19). In fact, sEPCR, can regulate the quantity of circulating aPC and influence the clotting of plasma (20). Ligand binding to EPCR promotes endocytosis of EPCR (21) via the Rab GTPase pathway (22). aPC/EPCR interaction can directly modulate cell signaling and alter gene expression in inflammation and apoptosis (23) and also provide cytoprotection via PAR-1 activation (24).

We previously demonstrated the expression of EPCR in a large number of cancer cell lines, in solid tumors (25) and malignant hemophaties. This led us to suggest that plasmatic sEPCR can be considered as a biomarker of cancer-associated hypercoagulability in human hematologic malignancies (26).

In the present study, we investigated the role of aPC, known to be a natural physiological anticoagulant, in the activation of cancer cells and the loss of clotting properties of ascitic fluid in patients with peritoneal ovarian carcinomatosis.

## Materials and methods

### Cells

The human ovarian cancer cell line OVCAR-3NIH, purchased from American Type Culture Collection (ATTC; Manassas, VA, USA), was cultured in RPMI-1640 medium containing 10% fetal calf serum (FCS), glutamine (03 mg/ml), penicillin (50 U/ml), streptomycin (50 *µ*g/ml) and incubated at 37°C with 5% CO_2_.

### Ascites

Peritoneal fluids were collected from 20 ovarian cancer patients treated at the Hospital Hôtel-Dieu (Paris, France). As evacuation of ascites is a part of the routine management of patients, only oral consent was obtained. Cells from ascitic fluids were pelleted by a short spin at 1,000 rpm, and the supernatant was re-centrifuged for another 10 min and then collected. The pelleted cells and the resulting supernatant were aliquoted and stored.

### Cyto-ELISA

OVCAR-3 cells (6×10^4^/well), in FCS-enriched medium, were seeded in flat-bottomed 96-well plates (Nunc). Confluent cell layers from 3-day cultures were washed with phosphate-buffered saline (PBS). The cells were then exposed to native protein C (Ceprotin^®^) or aPC (Xigris^®^) both at concentrations of 10 ng/ml for 2, 5 and 10 min. In parallel, culture medium with only FCS served as the control. The cells adhering to the plate were fixed with 100 *µ*l of 0.5 g/l glutaraldehyde and 0.4% Triton for 5 min at 4°C. Alternatively, the cells were fixed with 100 *µ*l of 100% ethanol for 5 min at −20°C. The plates were washed with PBS containing 0.02% Tween-20 and 10 g/l BSA (PBS-T-Alb), and then (5-wells/test) were exposed for 90 min to mouse anti-phosphorylated threonine or tyrosine antibodies (1/100 diluted in PBS-T-ALb). The immune complexes were detected by peroxidase-conjugated anti-mouse Ig (1:2,000). The fixed peroxidase was revealed by *o*-phenylenediamine benzidine (OPD) (0.2 g/l in 0.05 mol/l phosphate buffer, pH 5.0, containing 0.5 ml/l H_2_O_2_). The absorbance was measured at 492 nm. Control wells were constructed with isotype or without cells. Between each step, the wells were washed three times with PBS.

### Wound healing assay

OVCAR-3 cells (6×10^4^) were cultured in the presence of RPMI-1640 containing 10% FCS in 24-well plates coated with 0.2% gelatin. After 18 h, the semi-confluent cells were dislodged by a cell scraper on a standardized surface as previously described (27). The cells were then incubated in RPMI-1640 containing only 2% FCS (to reduce cell proliferation) in the presence or absence of PC or aPC (10 ng/ml). The effect of protein C on cell migration was evaluated (Microvision Instruments, Evry, France) by measuring the number of cells migrating to the wound edge after 6, 18, 27 and 48 h.

### Droplet test

To elucidate the mechanism of cell migration, we developed a ‘droplet model’ of Matrigel for the OVCAR-3 cells. The cell-Matrigel suspension (droplet) mimics an *ex vivo* cell cluster implant on the peritoneal membrane surface.

The ovarian cancer cells were incorporated in ice-cold Matrigel^®^ (50,000 cells/200 *µ*l Matrigel) to which was added either native or aPC at concentrations of 10 ng, respectively. A droplet of 10 *µ*l Matrigel-cell suspension was then delivered into each well of a 96-well microplate previously coated with 0.2% of gelatin and cooled down until the gel had solidified. Then, 0.2 ml of culture medium containing 2% FCS was gently added to the wells.

We studied the effect of 4 signaling inhibitors including 3 inhibitors of Ras signaling [UO126 (a MEK-1/2 kinase inhibitor), PD98059 (an ERK inhibitor) and FTI-277 (a farnesyl transferase inhibitor)] and one inhibitor of Rho GTPase (GGTI-298, ageranyl-geranyl transferase inhibitor). All inhibitors were from Calbiochem (France) and were used at a final concentration of 10 *µ*M added to the OVCAR cell suspension 15 min before adding the Matrigel. The number of cells migrating out of the droplet were observed and found to be time-dependent. They were counted after 18 h.

### Cell cycle (sub-G1) evaluation

OVCAR-3 cells were seeded in culture flasks and incubated with PC or aPC at 10 ng/ml for 24 h. Phase distribution (G1, S and G2) was performed using DNA content using flow cytometry and analyzed by Multi Cycle AV software as previously described (28). The coefficient of variation (CV) of the mean value of DNA-associated fluorescence of the G1 population (width of the peak) is a reflection of the accuracy of the DNA content measurement.

### Anticoagulant activity of living cells

Activated partial thromboplastin time (aPTT) evaluates intrinsic coagulation pathway efficiency; coagulation of a plasma sample is measured after addition of phospholipid (cephalin) and calcium. We previously described a method, based on aPTT, to estimate EPCR expression by endothelial and cancer cells (29), i.e. cells previously incubated with APC were added to a plasma and activated partial thromboplastin clotting time was measured. Cells expressing EPCR bind aPC, inducing a prolongation of aPTT. OVCAR-3 cells were seeded in 24-well macroplates, and after 24 h the cells were washed with RPMI-1640 and incubated with the addition of 50 *µ*g/ml aPC for 15 min at 4°C. After a further washing, the effects of OVCAR-3 cells (either remaining attached to the Petri dish or detached by Accutase) were tested on the cephalin clotting time of normal plasma.

OVCAR-3 (10×10^4^) cells were added to 0.1 ml normal plasma. After an incubation period of 3 min at 37°C, cephalin and CaCl_2_ were added to trigger coagulation. The clotting time was then measured at 37°C by a spectrophotometer at 595 nm. The results are presented both in real-time and as the ratio of cells + aPC/cells + PC.

### Determination of sEPCR, D-dimer and soluble fibrin (SF) in the ascitic fluid

sEPCR antigen was measured by Asserachrom sEPCR-ELISA immunoassay as recommended by the commercial supplier (Diagnostic Stago, Inc.). Fibrin degradation product, D-dimer concentration, was determined by STA^®^-Liatest^®^ D-Di, and SF monomers were quantified as previously reported (30).

## Results

The results that follow focus on the role of aPC in ovarian cancer cell activation as well as a loss of clotting observed for ascitic fluids. The investigation concerned several issues focused on the following:

### Evaluation of sEPCR and fibrin degradation products in the peritoneal fluid of ovarian cancer patients

All the samples tested (n=20) contained sEPCR. Eighty-five percent of the peritoneal fluid had an sEPCR concentration of 71±23 ng/ml which was lower than that of normal plasma (baseline values 100±28 ng/ml) (6). The remaining 15% samples revealed a sEPCR concentration which was similar to that of normal plasma (Table I). In parallel, D-dimer, a fibrin degradation product and SF were quantified (Table I). The 20 patients studied showed values for D-dimer (60%) and for SF (50%) which were superior to baseline values (500 ng/ml for D-dimer and 250 ng/ml for SF). The results presented in Table I indicated that in the peritoneal fluids there were ongoing processes of coagulation followed by fibrinolysis.

### Protein C induces cell cycle activation

**Figure 1:**
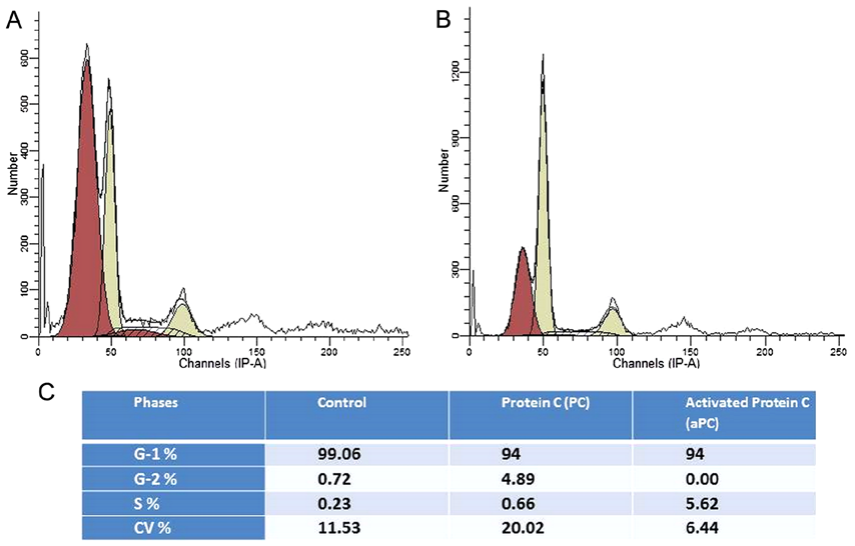
Influence of PC and aPC on cell cycle distribution of OVCAR-3 cells. Cell cycle (sub-G1) was analyzed by flow cytometry induced by PC (A) or aPC (B). Detailed results are presented in C. PC, protein C; aPC, active protein C; OVCAR-3, ovarian cancer cell line.

When cancer cells were cultured in medium containing 1% of FCS for 24 h, all cells entered into the G1 phase. Upon addition of culture medium containing protein C, 4.8% of the cells in the G1 phase underwent transition to the G2 phase (Fig. 1A), whereas when aPC was added, 5.62% of the cells in the G1 phase entered into the S phase (Fig. 1B). These results suggest that protein C influences the ovarian cancer cell cycle.

### aPC induces OVCAR-3 cell migration

**Figure 2:**
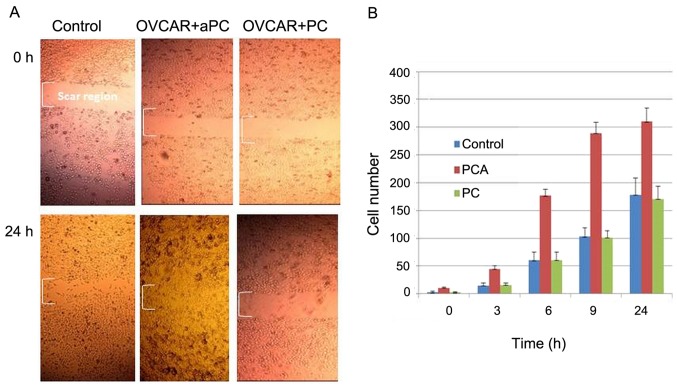
Influence of aPC and PC on ovarian cancer cell migration. (A) Results after 24 h compared with 0 h. (B) An increase in cell migration was induced by aPC at all time-lapses of the experiment. PC, protein C; aPC, active protein C.

The influence of protein C on OVCAR-3 cell migration at 3, 6, 9 and 24 h is presented in Fig. 2. It was observed that aPC enhanced cell migration into the wound, whereas in the presence of PC, cell migration was found to be similar to that of the control. The histogram in Fig. 2B depicts the effect of aPC and PC on ovarian cancer cells migrating into the scar region as a function of time (0, 3, 6, 9 and 24 h).

### aPC induces threonine and tyrosine phosphorylation of the OVCAR-3 cells

**Figure 3:**
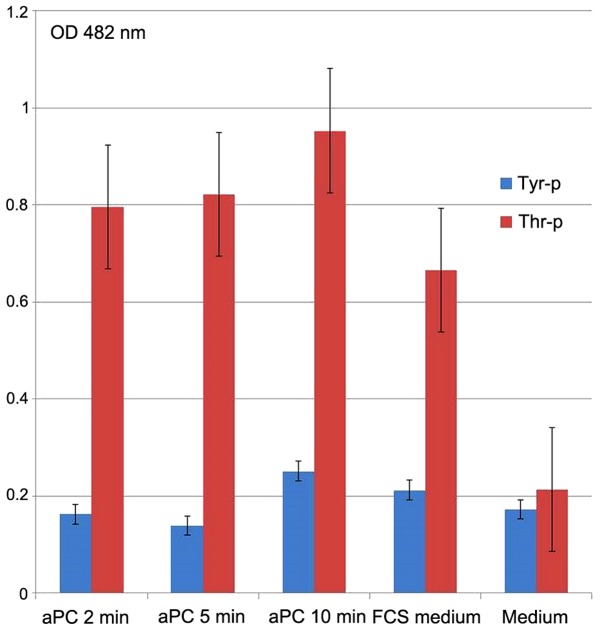
aPC induces threonine phosphorylation of the OVCAR-3 cells. Tyr-P, phosphorylated tyrosine; Thr-p, phosphorylated threonine. aPC, active protein C.

The effect of aPC on threonine and tyrosine phosphorylation in the OVCAR-3 cancer cells was tested by cyto-ELISA (Fig. 3). Compared to tyrosine, threonine was strongly and significantly phosphorylated when the cancer cells were incubated (10 *µ*g/ml) with aPC. Threonine phosphorylation started 2 min after aPC incubation, while tyrosine phosphorylation occurred later, that is only after 10 min of incubation. The medium with 10% bovine calf serum served as the control and was found to induce threonine and tyrosine phosphorylation.

### aPC upregulates cell migration via MEK-ERK and Rho-GTPase pathways in OVCAR-3 cells

**Figure 4:**
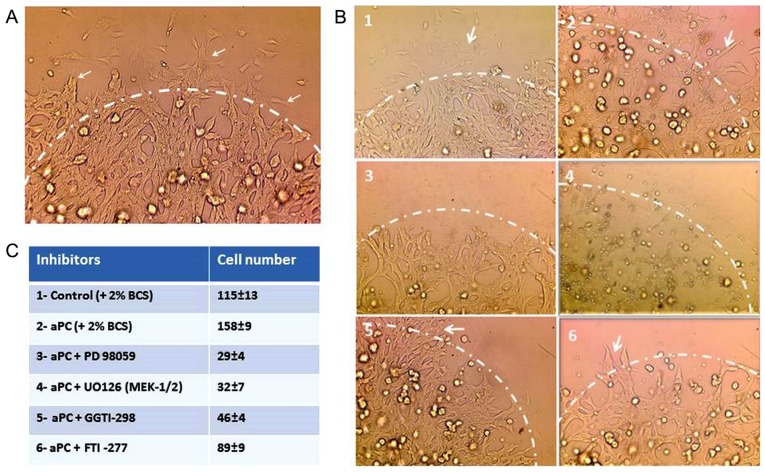
Cell migration in the droplet test (A) and the influence of several inhibitors of cell signaling pathways on the OVCAR-3 cells following treatment with the control (B1), aPC (B2), PD98059 an ERK inhibitor (B3), UO126 an MEK-1/2 kinase inhibitor (B4), GGTI-298 an inhibitor of Rho GTPases (B5), FTI-277 an inhibitor of farnesyl transferase (B6) on aPC-induced cell migration from the droplet were analyzed and quantified (C). aPC, active protein C.

The effect of inhibitors of several signaling pathways on cell migration was tested by droplet test (Fig. 4A and B). As presented in Fig. 4A, the cells were incorporated in the droplet. The cell migration from the droplet started after 2–3 h towards the outer periphery of the droplets (arrows). The outer of the periphery is indicated for clarity by a discontinuous line drawing. Fig. 4B indicates the control OVCAR-3 cells (Fig. 4B1) and the OVCAR-3 cells treated with aPC (Fig. 4B2). The cancer cell migration was inhibited by PD 98059 an ERK inhibitor (Fig. 4B3), UO126 an MEK-1/2 kinase inhibitor (Fig. 4B4) and GGTI-298 an inhibitor of Rho GTPases (Fig. 4B5), whereas the migration was unaffected by FTI-277 an inhibitor of farnesyl transferase (Fig. 4B6) and rapamycin a raptor-mTor complex inhibitor (data not shown). The number of cells migrating outside the droplets, in five experiments, is presented in Fig. 4C.

### Anticoagulant activity induced by OVCAR-3 cells

**Figure 5:**
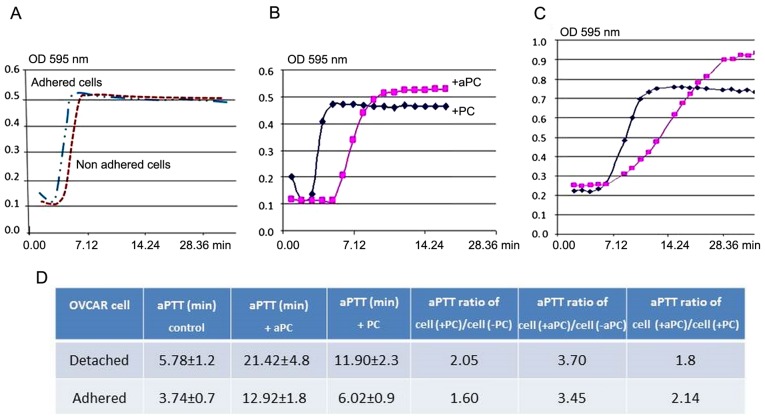
Fibrin formation kinetics on plasma in the presence of PC or aPC bound to OVCAR-3 cells tested by aPTT test. (A) aPTT test of adherent and detached OVCAR-3 cells without PC or aPC as controls. aPTT test of OVCAR-3 cells in (B) detached and (C) adherent conditions, in the presence of PC or aPC. (D) Real-time of aPTT (minutes) in different conditions. OVCAR-3, ovarian cancer cell line; PC, protein C; aPC, active protein C; aPTT, activated partial thromboplastin time.

The anticoagulant property of OVCAR-3 cells was assessed by measuring the prolongation of aPTT of normal plasma induced by aPC. As shown in Fig. 5A, the effect of OVCAR-3 cells, detached and incubated either with PC or APC, in fibrin polymerization curve of normal plasma is shown. The same experiment was also performed using OVCAR-3 cells in adherent conditions. When both detached (Fig. 5B) and adherent (Fig. 5C) cells, were incubated with aPC, aPTT was prolonged as compared to cells incubated with protein C. As documented in Fig. 5D, PC added to the adherent or detached ovarian cancer cells induced a 2-fold increase in cephalin clotting time, whereas aPC under the same conditions induced a 3.7-fold increase (5.78–11.90 min for PC and 21.42 min for aPC). These results indicate the anti-coagulant action of EPCR-aPC on the OVCAR-3 cells.

## Discussion

The peritoneal cavity is a space between the visceral peritoneum and the parietal peritoneum. Ten percent of all cases of ascites are of cancer origin (31). It is an exudate commonly observed at the late stage of cancer. Ascites may originate in the peritoneum (carcinomatosis) or may come from cancer that has spread from another site of the body. In ovarian carcinomatosis, the cancer cells adhere to mesenteric cells and form micronodules on the peritoneum (32).

Peritoneal fluids contain, besides cells, several proteolytic enzymes including metalloproteases and serine proteases (33). These fibrinolytic enzymes provoke increased extracellular matrix degradation and facilitate tumor cell invasion and metastasis (34) and as a consequence detach malignant cells which leave a maternal nodule for forming a secondary nodule on the peritoneal surface. Hence, considerable diminution of clotting of the peritoneal fluid plays a major role in the pathology and poor prognosis of ovarian carcinomatosis. We previously demonstrated that EPCR on endothelial cells has a physiological anticoagulant activity as ascertained *in vitro* by aPTT test (29). In contrast sEPCR, by its ability to trap aPC from plasma, can be considered as a cancer-associated hypercoagulability factor (26).

Cell migration was found to be inhibited when a neutralizing antibody against the EPCR antibody was added to the culture medium. In addition, we also showed that cell migration, induced by the binding of aPC to EPCR, was blocked by anti-ERK, MEK-1/2, and Rho-GTPase inhibitors when they were added while performing the droplet test. Our results indicate that the ERK-MEK-1/2 and Rho-GTPase signaling pathways significantly participate in the aPC/EPCR-PAR-1 induced cell migration. We found that the droplet test was a useful and informative model for studying cell migration. In addition, in another set of experiments, we also found that aPC-EPCR interaction increased cancer cell adhesion on the bottom of gelatin-coated culture flasks (data not shown). Here, we showed that the interaction of aPC-EPCR in ovarian cancer cells resulted in accelerated cell migration as evaluated by the kinetics of the wound closure.

When cells were synchronized and arrested in the G1 phase, their incubation with protein C or aPC induced cell cycle activation and passage from G1 to S or G2 phases after 18 h. These results on the activation of the cell cycle are in good concordance with our previous observation showing that aPC induces OVCAR cell proliferation (6). In a similar approach, but using human keratinocytes, Xue *et al* (34) showed that aPC stimulated the proliferation, migration and wound closure (35) again confirming that protein C induces enhanced cell migration.

The protein C system participates in the degradation of factors Va and VIIIa (9) thereby inhibiting fibrin formation. It also induces inhibition of plasminogen activator inhibitor-1 (PAI-1) (36). In order to estimate the ability of EPCR to bind aPC on the surface of living endothelial cells *in vitro* (29) we used a method that we had previously developed, based on prolongation of cephalin clotting time of plasma when aPC bound cells are added. This aPTT-based method was optimized to assess EPCR presence and functionality on the OVCAR-3 cell membrane. Our results showed that aPC bound on living ovarian cancer cells induced a prolongation of plasma clotting time suggesting that ovarian cancer cells use physiological anticoagulants such as aPC for their homeostasis.

EPCR exists as a membrane-bound form as well as a free sEPCR form. In fact, sEPCR can regulate the quantity of circulating aPC (20). Curiously, the sEPCR level in the peritoneal fluid of 85% patients was less than that in the plasma of healthy individuals. Only 3 patients (15%) had elevated levels of sEPCR (247, 250 and 154 ng/ml) which was below the level observed in plasma of patients with ovarian cancer (25). This indicates that sEPCR availability for trapping aPC is considerably reduced. Therefore, in ascitic fluids from ovarian cancer, free protein C binds to membrane EPCR of ovarian cancer cells, inducing cell migration ensuring the unclottability of peritoneal fluid by inhibition of the fibrin formation pathway.

Evaluation of D-dimer and SF in the peritoneal fluids of the ovarian cancer patients indicated that as soon as fibrin was formed, it was degraded. Moreover, the presence of EPCR-containing cancer cells in the peritoneal fluid limited the formation of fibrin on the cell surface as deduced from our observation indicating a marked increase in the cephalin clotting time of plasma.

In the peritoneal cavity, under other circumstances, aPC/EPCR interaction and cell activation can occur independent of the presence of cancer cells. There are a number of reports indicating that aPC/EPCR interaction via PAR-1 activation induces anti-inflammatory activity and anti-apoptotic activity (37,38). Peritoneal carcinomatosis is an inflammatory process and involves numerous non-tumor cells such as inflammatory cells. Inflammatory monocytes and neutrophils express EPCR on their membranes (17). The interaction of aPC/EPCR can downregulate the pro-coagulant activity of these cells. It can also induce cancer cytoprotection and enhance the malignant phenotype of cancer cells. The secretion of hyaloronan by mesenteric cells contributes to lubrication of the luminal peritoneal cavity. Whether EPCR is present or not on these cells has not been reported to date.

In conclusion, in ovarian carcinomatosis, aPC-EPCR interaction renders cancer cells highly aggressive, and as a result of inhibition of fibrin formation, the development of secondary nodules is facilitated. We are at present engaged in studies to further elucidate the role of EPCR in cancer homeostasis.

## Figures and Tables

**Table I:** Samples with an sEPCR concentration similar to that of normal plasma.

Samples	D-Di (ng/ml)	SF (ng/ml)	EPCR (ng/ml)
N.1	710	770	44
N.2	420	100	57
N.3	220	10	22
N.4	350	260	43
N.5	980	710	57
N.6	2,310	210	61
N.7	290	590	247
N.8	3,760	2,280	68
N.9	510	190	96
N.10	780	240	83
N.11	470	1,210	93
N.12	760	160	106
N.13	7,440	2,240	92
N.14	810	190	72
N.15	1,250	420	250
N.16	2,410	700	154
N.17	440	720	80
N.18	250	60	91
N.19	760	70	56
N.20	1,560	440	85

SF, soluble fibrin; EPCR, endothelial protein C receptor.
